# Sinus Floor Elevation via an Osteotome Technique without Biomaterials

**DOI:** 10.3390/ijerph18031103

**Published:** 2021-01-27

**Authors:** Rodrigo Andrés-García, José Vicente Ríos-Santos, Mariano Herrero-Climent, Pedro Bullón, Javier Fernández-Farhall, Alberto Gómez-Menchero, Ana Fernández-Palacín, Blanca Ríos-Carrasco

**Affiliations:** 1 Department of Surgery, Faculty of Medicine, University of Salamanca, 37007 Salamanca, Spain; randresgarcia@gmail.com; 2 Department of Periodontology, School of Dentistry, Universidad de Sevilla, 41009 Sevilla, Spain; pbullon@us.es (P.B.); alberto@gomezmenchero.com (A.G.-M.); brios@us.es (B.R.-C.); 3 Porto Dental Institute, 4150-518 Porto, Portugal; dr.herrero@herrerocliment.com; 4 Department of Periodontology, University Rey Juan Carlos, 28922 Alcorcón-Madrid, Spain; javier.fernandez@urjc.es; 5 Department of Social and Health Sciences, Universidad de Sevilla, 41009 Sevilla, Spain; afp@us.es

**Keywords:** sinus elevation, dental implants, osteotome, summers technique

## Abstract

According to classic Hirschfeld studies, the first teeth to be lost are the first and second maxillary molars. After the teeth are extracted and the alveolar process is developed, the maxillary sinus is reabsorbed and pneumatized with a decrease in bone availability in the posterior sector of the maxilla. This process often creates the need to perform regeneration techniques for the placement of implants in this area due to the low availability of bone. The most frequently used and documented technique for the elevation of the sinus maxillary floor is elevation by the side window, as proposed by Tatum. In 1994, Summers proposed a technique that allowed the elevation of the sinus floor from a crestal access using an instrument called an osteotome, as well as the placement of the implant in the same surgical act. The aimed of the study was to evaluate the survival of 32 implants placed in posterior maxilla with bone availability less than 5 mm performing a sinus lift augmentation technique with osteotome without biomaterials. The results of this study show a survival rate of 100% for 32 implants placed in situations with an initial bone availability of 2 to 5 mm without the use of graft material. The infra-drilling technique used offers an increase in the primary stability of implants that allows adequate osteointegration Implants placed were charged at 12 weeks. In all cases, spontaneous bone formation was observed, even in cases where a positive Valsalva maneuver was observed. This proposed technique reduces treatment time and the need for more invasive maxillary sinus augmentation techniques.

## 1. Introduction

According to the classic Hirschfeld study, the first teeth to be lost due to periodontal disease are the first and second maxillary molars [1]. After the posterior teeth are lost, the alveolar process is remodeled leading to alveolar bone resorption and pneumatization of the maxillary sinus resulting in reduced bone height in the posterior maxilla [2,3,4]. These changes often require regenerative techniques prior to any attempt at placement of implants in this area.

The most frequently used and documented technique for the elevation of the maxillary sinus floor is the lateral window osteotomy as proposed by Tatum [5]. Boyne and James proposed the additional use of biomaterials to elevate the Schneiderian membrane and promote bone formation [6].

Some disadvantages of this technique are the associated morbidity, the required healing period for ossification of the biomaterials, ranging from 6 to 10 months, and an additional second surgery for implant placement [7]. However, in some cases the implants can be placed simultaneously [8].

In 1994, Summers proposed a technique that allowed for the elevation of the sinus floor from a crestal access using an instrument called osteotome, along with immediate implant placement [9,10]. This technique is less invasive and reduces treatment duration. One of the initial limitations of this technique is the minimum amount of bone height needed ranging from 4 to 6 mm to achieve adequate primary stability [11].

Over the years, the macroscopic design of dental implants has evolved, changing to a conical shape with a shorter distance between threads, as well as a tendency towards a conical connection. With these changes, greater primary stability can be achieved in areas of low bone availability [12,13,14]. Moreover, studies have emerged where implants were placed in the posterior area of the maxillary region in cases of ≤4 mm of bone height with a crestal access and no statistically significant differences compared to the conventional technique were found [15,16,17].

Nedir proposed the placement of 10 mm implants in areas of 6 mm bone height using a conventional osteotome technique without the use of any regenerative biomaterials. Over time, the author observed spontaneous bone formation around the apex of the implants, with an implant survival rate of 100% during a 3-year follow-up. In addition, the stability of the newly formed bone was confirmed [18]. Recent publications have compared the sinus floor elevation with and without the use of biomaterials in terms of implant survival and bone formation, and no statistically significant differences have been found [19]. The spontaneous bone formation maybe be due to a phenomenon called “Periosteal guided bone regeneration” first described by Lundgren where the sinus membrane is elevated and maintained on top of the apex of the implants, or by suturing, without biomaterials, generating a space which is later filled with a blood clot and subsequently colonized by osteoblasts from the adjacent bony walls of the maxillary sinus as well as from the Schneiderian membrane [20].

In 2002, Fugazzotto developed a technique for sinus augmentation performing a crestal access using a drill and a calibrated osteotome. This osteotome is one drill size narrower than the normal site preparation and no biomaterials are used. This technique showed a survival rate of 98.3% after a 4-year follow-up. The author developed a formula to help choose the length of the implant to be placed, the 2X-2 rule, where “X” represents the residual bone coronal to the floor of the sinus. The result of this formula is the length of the implant to be placed [21].

Few articles have assessed the presence/absence of Schneiderian membrane perforation after sinus floor elevation performed with a crestal access, and the relationship between the bone formation after osteotome elevation procedures [22,23,24,25,26,27]. A tear in the Schneiderian membrane can complicate sinus floor augmentation and depending on the size of the tear the placement of a filler biomaterial may be contraindicated.

Maxillary sinus floor anatomy is not homogeneous. Implants placed with osteotomes in close vicinity of the mesial or distal wall of the sinus or in contact with an Underwood septum could behave differently. One possible advantage is the spontaneous bone formation around the apex of the implants. The space created under the implant-created-tent is filled with a blood clot rich in osteoblasts allowing for new bone formation.

The present study aimed to evaluate the survival rate of 32 implants placed in the posterior maxillary region in locations where bone availability ranged from 2 to 5 mm. A trephine drill was used for the preparation of the implant site and no biomaterials were used. The newly formed bone around the apex of the implants was measured during an 18-month follow-up period. At the same time, the incidence of Schneider membrane tears and spontaneous bone formation was investigated.

## 2. Materials and Methods

This study was approved on 23 June 2011 by the ethics committee of the Faculty of Dentistry of the University of Seville (Spain). All patients were informed about the type of study in which they were going to be included, as well as the advantages and disadvantages of the surgery to which they would be subjected. Thirty-two patients were selected from the Master’s Degree in Periodontics and Implants of the Faculty of Dentistry of the University of Seville (Spain).

The inclusion criteria were as follows:

(a) Patients older than 18 years of age with good general health status
(b) Absence of one or more teeth in locations where bone availability to the sinus floor was between 2–5 mm;
(c) At least 4 months of healing had occurred between tooth extraction and implant assessment;
(d) After implant placement the bone around the entire implant shoulder should be ≥1 mm;
(e) No removable prostheses;
(f) The patient had to sign an informed consent and accept to return for review appointments.

The exclusion criteria were as follows:

(a) Patients with severe bruxism;
(b) Smokers of more than 10 cigarettes/day;
(c) Local, acute or chronic sinus pathology;
(d) Presence of Underwood septa in the implant area.

### 2.1. Surgical Sequence and Prosthetic Rehabilitation

As a guideline for pre-surgical antibiotic prophylaxis, patients were prescribed 2 g of amoxicillin 1 h before surgery or 600 mg of clindamycin in cases of amoxicillin allergy. The technique used was a modified version of the one proposed by Fugazzotto in 2002 [21]. Under local anesthesia a full-thickness flap was raised so the entire bone crest could be observed. A single trephine drill was used at 400 rpm for preparation in order to try to discriminate against the change in resistance that occurs when reaching the cortical sinus floor. The trephine drill selected had an external diameter of 3.5 mm when implants had a 4.3 mm diameter or 4 mm in cases where implants had a diameter of 5 mm. Moreover, we tried to maintain a bony cylinder in the drilling area. In cases where the bone cylinder was fractured or retained in the trephine, it was not used as a filler. The preparation length was 1 mm shorter than the initial bone height, e.g., if we had 4 mm of available bone, the trephine was used to 3 mm. Subsequently, a Camlog^®^ osteotome (Camlog, Wimsheim, Germany) was chosen. This instrument had a diameter immediately smaller than the diameter of the implant used to fracture the sinus floor. Valsalva maneuver was performed to assess the integrity of the Schneiderian membrane. Regardless of the Valsalva maneuver being positive or negative, the implant was placed. The delimited bone cylinder normally undergoes compression of the medullar bone between the cortical area of the bone crest and the cortical area of the sinus floor. This cylinder was impacted in an apical direction to facilitate the elevation of the membrane. Implants were placed at 30 rpm until the entire rough surface was buried. All implants used were from the Camlog^®^ Promote Plus^®^, Screw-line product line. All implants were 9 mm long and the diameter varied between 4.3 mm and 5 mm. The final step was the placement of a cover screw to allow for a submerged healing and flaps were sutured using a 4–0 polyamide suture.

The postoperative instructions were amoxicillin 500 mg every 8 h for 7 days or clindamycin 300 mg every 8 h for 7 days in cases of penicillin allergy; ibuprofen 600 mg every 8 h for 4 days, and 0.12% chlorhexidine rinse twice a day for 14 days.

### 2.2. X-ray Measurements

The available bone at the beginning of the study, as well as the evaluation of the newly formed bone at the different follow-up visits, were measured with periapical X-rays obtained with parallelization devices (Rinn XCP rings. Dentsplay Sirona Inc., Delaware, PA, USA). In order to standardize X-ray projections, bite blocks with heavy body silicone (Aquasil Ultra Putty Soft Regular Set, Dentsply Sirona Inc.) covering the devices were prepared for each patient. As the implant length was known and once the measurements were made, a rule of three was used to discard possible magnifications of the image. All measurements were done by the same clinician, who also marked the measurement dots on the X-rays. Measurements were made three times to confirm the lengths. The reference points were: (A) coronal reference point (independent of the bone crest): the most coronal contact between the bone crest and implant (CO) or the implant shoulder itself (HI). (B) apical reference point: at baseline, the most apical contact between the implant and the original sinus floor (CS1) and at the 18-moth follow up, the most apical contact point between the implant and the newly formed cortical sinus (CS2).

**Figure 1:**
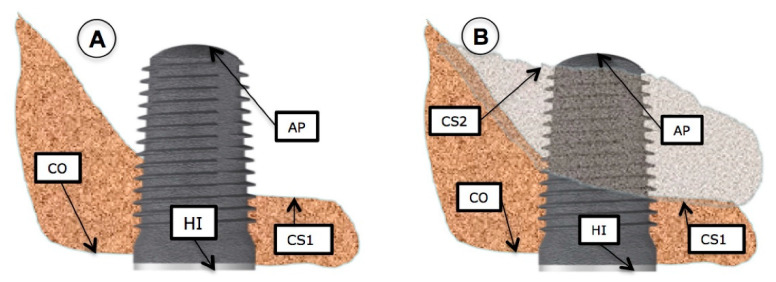
(**A**) Situation immediately after implant surgery. CO: Bone crest. HI: Implant shoulder. CS1: Baseline cortical sinus floor. AP: implant apex. (**B**): 18-month follow-up newly formed sinus floor (CS2) can be observed.

**Figure 2:**
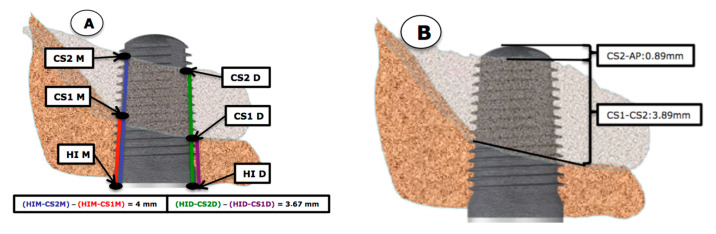
(**A**) Representative measurements made at the mesial and distal aspects of the implant. You can see how newly formed bone at the mesial and distal aspects were obtained by subtracting HIM–CS1M from HIM–CS2M to determine the mesial gain, and the distal gain was determined by subtracting HID–CS1D from HID–CS2). (**B**): Representation of the bone gain obtained measured from the baseline sinus floor to the newly formed sinus floor cortical bone (CS1–CS2), as well as the uncovered implant apex protruding into the sinus floor.

**Figure 3:**
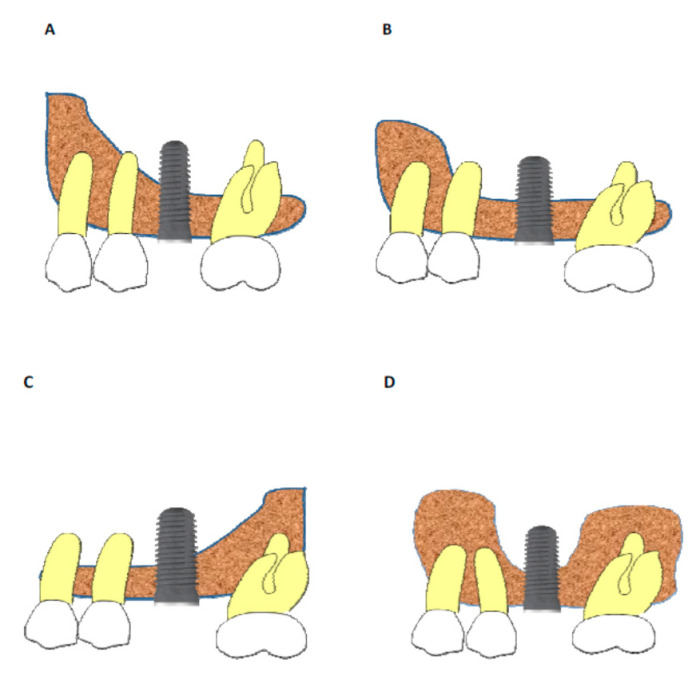
Schematic representation based on implant locations relative to the maxillary sinus anatomy. (**A**) The implant is in close vicinity to the mesial wall of the maxillary sinus; (**B**) The implant is located in a flat area in the maxillary sinus; (**C**) The implant is in close vicinity to the distal wall of the maxillary sinus; (**D**) The implant is in contact with both mesial and distal maxillary walls (e.g., narrow maxillary sinus) or is in close contact with an Underwood septum.

The measurements were made at the mesial, middle and distal sites. To determine the amount of bone gain at the mesial area of the implant apex, firstly the distance from the most mesial area of the implant shoulder (HIM) to the most apical and mesial contact point of the newly formed cortical bone around the implant apex was evaluated after 18 months (HIM–CS2M). This measurement was then deducted from the baseline value which was the distance from the most mesial point of the implant shoulder (HIM) to the most apical and mesial implant contact point with the baseline sinus floor (CS1-M) A calculation was made where the distance HIM–CS1M was subtracted from HIM–CS2M. This positive value would be the spontaneous bone gain obtained in the mesial zone. For distal measurements, the same measurements were made, but at the distal location, which was the result of the subtraction (HID–CS2D) − (HID–CS1D). The same occurred at the medial zone: (HI–CS2) − (HI–CS1). This process is shown in Figure 1 and Figure 2. In cases where spontaneous bone formation occurred beyond the implant apex, the line (HI-CS2) was extended apically until it met CS2, which was sometimes more apical than implant apex (AP) as can be seen on X-ray B in Figure 3.

X-rays and initial assessment were performed one day before the surgery. The initial bone availability was measured at the center of the location. Bone height ranged from 2 to 5 mm. Subsequently, X-rays were taken at 3 months (second stage surgery) as well as at the 12- and 18-month follow-up. After the prosthesis delivery the individualized bite block devices were adapted to the new clinical situation so they could continue to be used.

### 2.3. Relationship between the Maxillary Sinus Cavity and Implant Location

**Figure 4:**
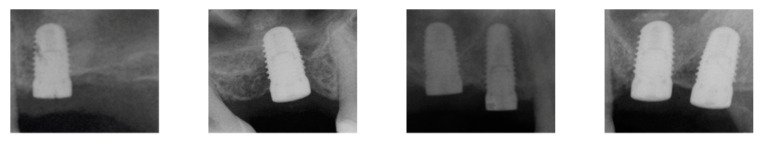
Bidimensional classification of implant locations relative to the maxillary sinus anatomy.

Our study also evaluated the differences in terms of bone gain around the implant apex in different anatomical situations and their relation to the maxillary sinus. Moreover, the location of the implant within the maxillary sinus cavity and the anatomy could be related to greater or lesser bone formation around the apex of the implants. This could be related to the space created under the ‘implant-created-tent’ allowing for new bone formation Likewise, depending on the proximity to osseous walls, the implants could be in direct contact with the mesial wall, with distal wall, with both of them or none of them. Therefore, the following anatomical classification of the maxillary sinus in cases of implants placed with the osteotome technique was proposed (Figure 3 and Figure 4):

Three months after implant placement, the second surgical stage was performed, replacing the cover screw for a 4 mm abutment. 2 weeks later, a conventional closed-tray implant impression technique was carried out. Finally, the definitive screw retained metal-ceramic restorations or screw-cement retained restoration was delivered using Camlog^®^ Esthomic. Provisional crowns were not needed. Once the crowns were screwed on the implants, the silicone bite blocks were adapted to be able to use in the same position as initially used. If X-rays were not possible to be taken, the crown was unscrewed and the X-ray was carried out without the crown.

In cases where a new occlusal scheme was needed, mutually protected occlusion was generated, similar contacts in maximum intercuspation, with anterior guidance and subsequent posterior disocclusion. The opposing dentition was not taken into consideration whether it was natural dentition, a single crown or removable prosthesis.

### 2.4. Statistical Analysis

Firstly, data cleansing was performed using numeric and graphical methods. Quantitative variables were summarized with typical mean values and deviations, and in the case of asymmetric distributions, medians and percentiles were used, as shown on *p* (25) and *p* (75). The descriptive analysis was completed with the corresponding graphical representations. Qualitative variables were expressed in percentages.

We then assessed the initial bone height, the distance between the implant shoulder and the baseline sinus cortical floor, the distance between the implant shoulder and the newly formed sinus floor in contact with the implant at the 18-month follow up, the distance between the baseline sinus floor cortical bone and the implant apex and finally the distance between the newly formed sinus floor cortical bone.

The following qualitative variables were evaluated: tooth type, implant diameter, implant length, prosthetic restoration, Valsalva maneuver, sinus anatomy and mesial sinus wall.

Subsequently, a new variable ‘Average gain’ ((mesial gain + distal gain)/2) was included, and its relationship with the Valsalva maneuver was determined using a Student T-test (the normality was checked with the Shapiro–Wilk test).

The Valsalva maneuver was correlated with the sinus anatomy (Chi square) and the sinus type was correlated with the mean gain (Kruskall–Wallis test was conducted for independent samples). A correlation was established between the Valsalva maneuver variable and the initial bone availability using the Mann–Whitney U-test after checking the normality with the Shapiro–Wilk test.

Finally, the relationship between the initial bone height distribution and the different sinus types (Kruskall–Wallis test) was analyzed after assessing the normality (Shapiro–Wilk test).

## 3. Results

The 18-month implant survival rate was 100%. No episodes of epistaxis or signs of infection were observed either at the maxillary sinus or at the implant sites. Furthermore, no complications beyond loosening of a healing abutment during the fabrication of the prosthesis occurred.

No complications were reported in relation to the actual sinus lift procedure (e.g., epistaxis, sinusitis). The average initial bone availability, measured at the center of the first drilling preparation, was 3.69 ± 0.89 mm, with a minimum of 2 mm and a maximum of 5 mm. The distance from the implant contact point to the bone crest (CO) and the mesial contact point between the implant and the sinus floor (CS1M) was 3.85 ± 1.15 mm (CO-CS1M), and 3.84 ± 1.27 mm (CO-CS1D) at the distal area (CS1D). Another measure was made mesially from the implant shoulder (HI) to the contact point of the implant with the sinus floor cortical bone resulting in 4.29 ± 0.9 mm (HI-CS1M). This measured 4.45 ± 1.09 mm at the distal aspect (HI-CS1D).

The use of the Valsalva maneuver to assess the possible tearing of the membrane gave a positive result in 8 (25%) of the 32 cases.

After spontaneous bone formation around the apex of the implants, a new maxillary sinus floor (CS2) was observed 12 months after surgery (Figure 1). This bone gain was measured from the HI-CS2—HI-CS1 distance, with an average result of 3.89 mm. Looking at these results at the mesial and distal aspects, the gain would be 4 mm in the mesial aspect and 3.67 mm in the distal aspect. In several cases spontaneous bone formation was seen apical to the implant apex. The mesial bone gain resulted statistically significant (*p* < 0.026) compared to the distal site. Spontaneous bone formation was observed to a greater or lesser extent in all cases assessed in the study (Figure 2).

The amount of uncovered implant in the apical area (AP) (AP-CS2) was 0.7 ± 1.69 mm mesially and 0.87 ± 1.47 mm distally with an average of 0.89 mm in the central area of the implant.

**Figure 5:**
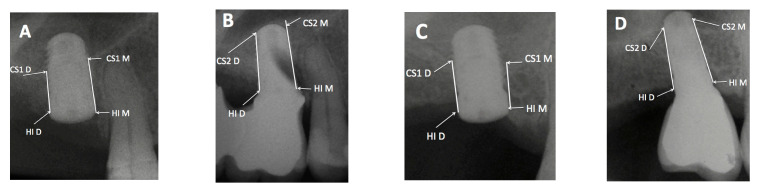
(**A**): Post-surgical periapical X-ray of Patient 1. (**B**): Periapical X-ray at 18 months of Patient 1. (**C**): Post-surgical periapical X-ray of patient 2. (**D**): Periapical X-ray at 18 months of patient 2. In all cases, bone gain was obtained between baseline (**A**,**C**) and the 18-month follow-up (**B**,**D**) can be seen.

**Figure 6:**
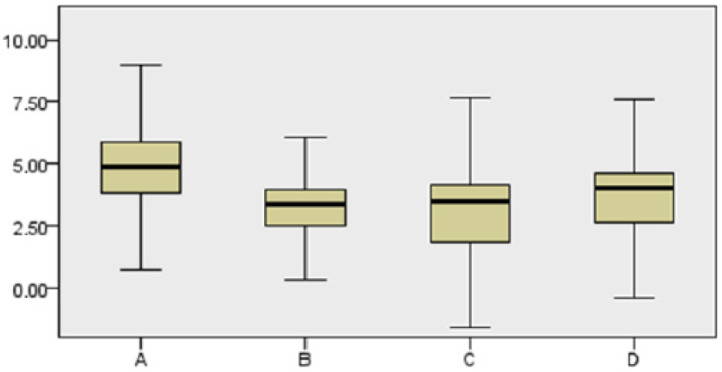
Bone gain ratio obtained based on the implant location according to the proposed classification (Sinus type).

Furthermore, we analyzed the relationship between the maxillary sinus anatomy and the position of the implant in terms of spontaneous bone formation, and a two-dimensional classification of four types is proposed (Figure 3, Figure 4, Figure 5 and Figure 6):

- Sinus type A: implant is close to the mesial wall of the sinus (25% of cases);
- Sinus type B: implant is located in a flat sinus floor at some distance from the osseous walls and/or partitions. This was the most common situation representing 37.5% of cases;
- Sinus type C: implant is in contact with, or very close, to the distal sinus wall (12.5% of cases);
- Sinus type D: implant is very close to a bony wall at the mesial and distal aspects of the maxillary sinus or in contact with an Underwood septum, delimiting the maxillary sinus into different compartments (25% of cases).

No statistically significant relationship was observed between sinus type and the possible finding of a positive Valsalva maneuver; however, there was a greater tendency for a positive Valsalva maneuver in type B situations (50% of cases). In the other types, the Valsalva maneuver was positive in 25% of the cases.

Our study compared the different types of the maxillary sinus in relation to spontaneous bone formation, and no statistically significant differences (*p* > 0.05) were found. Greater bone formation was observed in sinus type A, where the implant is close to the mesial wall of the maxillary sinus, although this difference was not statistically significant (Figure 6). No statistically significant differences were found in relation to initial bone availability and the presence of a positive Valsalva maneuver although there was a greater tendency for a positive Valsalva maneuver when baseline bone height was reduced.

## 4. Discussion

In 1987, Misch proposed a classification for the treatment of the edentulous posterior maxilla, based on the residual bone below the antrum. For more than 30 years the sinus augmentation techniques have been evolving [28]. Currently, for the management of the atrophic posterior maxilla, there is a tendency to perform more conservative and less invasive techniques, crestal access treatments and placement of shorter-length implants [15,16,17,18].

Studies have shown that implants placed using osteotomes have similar results compared to implants placed using a conventional technique [20,21,22]. A retrospective study by Rosen in 1999 included the placement of implants using Summers original technique. This author concluded that when there was ≥5 mm of bone height the results obtained were similar to those placed in conventional locations [11].

The article published by Fugazzotto, in 2002, introduced a trephine technique combined with osteotomes in locations of 4–5 mm of bone height obtaining a survival rate of 98.5%. The technique described in our material and methods was based on Fugazzotto’s osteotome technique [21]. The underpreparation was performed with an approximately 0.8 to 1 mm discrepancy between the implant diameter and the implant bed, obtaining an insertion torque >20 N/cm in every case. In addition, 100% of the implants achieved osseointegration. The subjective clinical perception in terms of enhanced primary stability was positive and the use of a single trephine facilitates the axial implant preparation site.

A 5-year randomized clinical trial published by Cannizaro in 2013 compared 8 mm implants placed with osteotomes versus longer implants placed with a lateral window sinus lift procedure, in locations of 3–6 mm bone availability. The results indicated no differences between the techniques. Furthermore, the lateral window approach was associated with a higher complication rate including biomaterial infections and implant failures [29].

In a metanalysis published in 2018 by Al-Moraissi, the recommended treatment in cases of 4–8 mm available bone height in the posterior maxillae is osteotome sinus lift with immediate implant placement [30]. Furthermore, no statistically significant differences between the length of the implants and the survival of the implants was found.

Other authors have published similar outcomes in cases of ≤ 3 mm bone height with a survival rate ranging from 94% to 100% [31,32,33,34]. These authors stress the importance of “undersizing” the implant site in order to obtain correct primary stability. Placement of short implants in these situations have similar survival results resulting in 96% survival rates [35].

Bolle in 2018, compared the survival rate of 4 mm long implants versus 10 mm implants placed with open sinus lift procedures, reporting no significant differences. The author concluded that the option of short implants is preferable due to the lower occurrence of problems related to bone augmentation and graft techniques. This study had a 1-year follow up, which we understand is insufficient so longer-term studies are needed [36].

Taschieri et al. published several studies in 2017 and 2018 where they confirmed that the crestal sinus lift approach had less patient morbidity as smaller mucoperiosteal flaps are required when compared with the lateral window procedure resulting in a significantly better postoperative healing (e.g., less inflammation, less pain and faster recovery including normal daily activities) [7,37,38].

Leblebicioglu, in 2005, published an article where implants were placed using osteotomes without the use of grafting material finding a spontaneous bone gain of 3–4 mm [39]. Leblebicioglu was one of the first authors to perform sinus augmentation without the presence of any graft material. Many authors have continued this approach, obtaining similar results in terms of survival and spontaneous bone formation [15,16,17,19,20,24,27,33,34,40].

In a metanalysis published by Antonaya-Mira, Martínez and Moraschini, the osteotome technique without the use of grafting biomaterials reported average bone gains of 3.43 mm [24,41,42] thus validating this procedure.

Several articles have described the bone gain around the implant apex without concurrent use of any regenerative material due to the “tent” phenomenon [17,18,19,22,24]. This singular process is achieved by elevation (prior to fracturing the sinus floor with an osteotome) of a bony fragment. The Schneiderian membrane will follow this fractured bony fragment and will be the apex of the implant itself and is responsible for keeping the sinus membrane elevated like a tent. This space will be occupied by a blood clot that will be colonized by osteoblasts over time. The results of previous studies show a non-statistically significant tendency for increased bone formation when biomaterials are used compared to non-grafted sites. However, and over time, these differences were minimal due to the reabsorption of part of the biomaterial placed in the grafted group and the progressive mineralization of the spontaneously formed bone in non-grafted sites [22,24,25,31,43].

Another explanation could be a direct role of the sinus membrane in the bone healing process. Along with osteogenic properties, the sinus membrane could also protect the blood clot during healing acting as a barrier membrane possibly explaining the spontaneous bone formation obtained in this study [20].

This newly formed bone undergoes progressive mineralization, a sign of stability over time, compared to pneumatization of the maxillary sinus seen after lateral window sinus augmentation procedures [44]. A biomaterial volume reduction of 25% has been observed after a healing period of 6 months [45]. In addition, Hatano showed that, over time, it was difficult to find implants that had the apex coated by the biomaterial even though they were initially completely covered [46].

In relation to the amount of newly formed bone around the implant apex, a complete fill of the apex was not observed in all cases, however, a partial coverage of the implant body that was initially apical to the original cortical floor of the maxillary sinus (CS1) was observed in every case. Complete fill occurred in 12 of the 32 cases (37.5%). In 12.5% of cases, 3 mm of the apical part of the implant remained uncovered. Statistically significant differences were found in relation to the mesial or distal area of the implant in favor of the mesial aspect (*p* < 0.05). Our radiographic findings suggest the sinus membrane covers completely the implants that protrude into the sinus <4 mm. In cases where this protrusion is >4 mm the sinus membrane made a partial seal, allowing for some of the implant to be left inside the maxillary sinus [47,48].

The most coronal reference point used was the implant shoulder. An osseous reference point was not used due to the crestal resorption process that may occur once the abutment and crown are placed on the implant [49].

One of the cases where the initial bone availability was 2 mm had a final bone gain of 4.5 mm after 30 months of follow-up. In this particular case the patient presented a positive Valsalva maneuver. The 4.5 mm osseointegrated implant surface appeared to have been sufficient for implant survival.

Sinus membrane tearing, identified by a positive Valsalva maneuver, was shown to have a negative effect on the formation of new bone, but this finding was not statistically significant. In the positive cases, less bone gain was obtained. The membrane tear rate found in this study was 20%, higher than the average percentage found for closed techniques, and more similar to open techniques [26,40].

These results may be due to the initial amount of bone height which was lower than reported in other studies or because the percentage of membrane tears was not mentioned in the majority of closed technique studies. Gabbert found Schneiderian membrane perforation rate of 26% but this had no impact on implant osseointegration or implant survival [50]. As the author stated, these membrane perforations are difficult to observe during preparation, but they are usually smaller compared to the tears that occur in open sinus techniques. The main problem of this procedure is that it is a blind technique and the only method to check membrane integrity is the Valsalva maneuver. In addition, as no graft material was used, there was no risk of penetrating into the sinus cavity. In general, studies using an osteotome technique with bone availability <4 mm have not mentioned membrane perforation rates [15,16,17,18,24,25]. In our study, the 20% of implants with positive Valsalva maneuver did not show lower levels of osseointegration and implant survival.

When there is limited bone height a greater elevation of the Schneiderian membrane is needed for implant placement. This membrane has an average elasticity of 5 mm [51,52,53]. This justifies for positive and negative Valsalva maneuver. None of the perforation cases had complications. These results are consistent with the literature [54]. Sun-Hang, in 2008 in a histological study observed that when the implant apex protruded less than 4 mm the Schneiderian membrane is able to completely seal itt. When the protrusion was >4 mm only partial healing was seen [54]. Jung, Tabrizi and other authors observed that when the implant apex protruded into the sinus membrane no pathology was seen beyond asymptomatic thickening of the sinus mucosa [27,55,56]. No episodes of ostium obstruction were reported. In our study during the 18-month follow-up no adverse episodes were detected.

The mean amount of bone gain was 3.89 mm, similar to the 3.9 mm obtained in the Nedir et al. study [23]. If we look at the differences in bone gain at the mesial and distal aspects of the implant apex, at the mesial area, the mean amount of bone formation was 4 mm, and at the distal area this gain was 3.67 mm. This difference was statistically significant. Probably, this difference occurred because the largest number of implants were placed in the mesial area of the maxillary sinus, close to the mesial limit. This could have favored a greater contribution of osteoblasts and clot stabilization.

A systematic review and meta-analysis published by Moraschini, reviewed 18 articles related to sinus augmentation procedures without grafting material obtaining a mean bone gain >4.7 mm and a survival rate 97% [24]. The systematic review included 15 studies where periapical and panoramic X-rays were used for the evaluation and measurement of the newly formed bone around the implant apex. Other authors such as Kovacs and Fornell [57,58] prefer Conical Beam Computer Tomography (CBCT) for initial assessment and treatment planing. For follow-ups and bone gain measurements, these same authors recommend periapical X-rays due to the good details obtained, less distortion and less metallic artifacts. Other advantages of periapical X-ray include usability and lower radiation especially in cases of follow-up measurements which are not directly involved in treatment planing.

The two-dimensional images only determine the gain at the mesial, medial and distal areas. Hage and coworkers evaluated the bone gain obtained around the apex of implants using CBCT, and observed that the bone gain obtained at the buccal and palatine aspects of the apex of these implants was at the same level compared to the mesial and distal aspects [59]. This confirms that measurements at the mesial and distal levels of two-dimensional images obtained from periapical X-rays are similar to those obtained in the buccal and palatine areas. Thus, in the absence of symptoms or complications of the maxillary sinus, it was considered as sufficient to perform periapical X-rays to assess the bone gain [60].

Numerous articles published by Nedir and coworkers, have evaluated implants with periapical X-ray techniques, being a reliable, simple, cheaper, useful, usable and with a lower radiation dose compared to other techniques [16,18,21,23,24,25].

Si et al. compared the use of crestal osteotome sinus elevations with and without grafting materials in Labrador dogs. After 24 weeks of healing, the bone density of the newly formed bone was 35.90% in the non-graft group vs. 25.59% in the graft group. They further found the amount of new bone was much higher in the non-grafted group than in the grafted group. Similarly, the percentage of bone-to-implant contact was almost double at 24 weeks in the non-grafted group (23% vs. 40%) [47].

Our results are consistent with those obtained by other authors [19,24]. There is enough scientific evidence to demonstrate the predictability of this technique including numerous systematic reviews and meta-analysis [24,30,41,42].

Every case was restored with either screw retained restorations or cemented-screw retained restorations. This allowed for retrieval in cases where it was not possible to adapt the individualized X-ray bite blocks. During the 18-month follow up, only two crowns had to be re-tightened, the occlusion was adjusted and no more complications were seen.

## 5. Conclusions

The results of this study show a survival rate of 100% for 32 implants placed in situations where the initial bone availability ranged from 2 to 5 mm without the placement of any grafting materials. The infra-drilling technique offers an enhanced primary stability allowing successful osteointegration. All implants were loaded after 12 weeks of healing, even in cases where the baseline bone height was 2 mm. All cases showed spontaneous bone formation including cases where a positive Valsalva maneuver was observed. This technique reduces the overall treatment time and reduces the need for more invasive maxillary sinus augmentation procedures.

## Data Availability

The data presented in this study are available on request from the corresponding author. The data are not publicly available due to privacy.
